# The New Sleeping Sickness

**Published:** 1919-05

**Authors:** 


### The New Sleeping Sickness.

Reports in the daily press of the occurrence of "sleeping sickness" in various parts of the United States have been assumed by many readers to refer to the well-known African malady of that name, believed to be communicable only by the bite of the tsetse fly, an insect unknown in this country. The present disease is apparently encephalitis lethargica, or lethargic brain-fever, an obscure condition which occurred in an epidemic form in England last year. From an account in *The Lancet* (London) of a very full discussion at the Royal Society of Medicine, it appears that the disease may cause simply a general disturbance of the nervous system, or may be localized in the brain, spinal cord, or elsewhere. It begins with lethargy and headache, lasting from one to three weeks and followed by stupor, delirium, pain, and paralysis. There are often serious after-effects, paralytic and other, and the disease appears in many ways to be closely related to infantile paralysis, altho not identical with it. With regard to the cause of the disease, we summarize the abstract of a committee report read at the meeting named above, made for *The Interstate Medical Journal* (St. Louis, April):

"Various hypotheses had been suggested by different workers engaged in the inquiry, but the views which appeared best to agree with present knowledge was that encephalitis lethargica was one of a group of diseases in which, as in cerebrospinal fever and acute poliomyelitis, the pathogenic agent was much more generally present in the human organism than the clinical evidence implied. As regards cerebrospinal fever, this was no longer a hypothesis, but a well-established observation. In that condition the specific reaction named cerebrospinal fever arose in one or other of two ways: first, as the result of a breakdown in the immunity to the effects of the virus which the individual who harbored it had up to that time enjoyed; secondly, as the result of a non-immune person becoming infected with a strain of the virus which had attained the degree of pathogenic action described as a specific.

"This was the view that best explained the irregular, widespread, sporadic distribution of encephalitis lethargica. Until further research yielded precise information, we might assume that many people harbored the organism of the illness—and that in certain of these persons there occurred for some unknown reason either an enhanced virulence of the parasite or a lowered resistance of the tissue cells, or both, the result being that the stimulus of the parasite overcame the resistance of the tissue cells and the host suffered from the effects of the virus which previously he had harbored with impunity. This explanation implied that the key to the problem rested not in the purely bacteriological view of the causation of disease, but in the wider view that disease resulted from the interaction of several factors.''

One suggestion of more than usual interest was that this and other nerve-diseases have been simply incidents of the influenza epidemic. We read:

''Dr. W. H. Hamer pointed out that, just as in 1915, the so-called epidemic of cerebrospinal fever was really only a small part of an influenza prevalence, so in the epidemic encephalitis of the spring of 1918 a particular cluster of symptoms was once more being singled out for scrutiny, and that here again the epidemic, so called, was merely part and parcel of a prevalence of influenza. During the last ten years evidence had been collected, however, clearly showing a close association between outbreaks of poliomyelitis, polioencephalitis, cerebrospinal meningitis, and prevalences of influenza. The proof that epidemic lethargy was no new disease was writ large. In view of the fact that 'Spanish influenza' had followed hard on the heels of epidemic encephalitis, he ventured to plead, once again, for the epidemiological point of view. These 'new diseases' coud not be completely understood by making cell counts and cultivating various strains of organisms; nor satisfactorily explained by focusing attention on some particular sign or symptom, whether it were sweat, lethargy, paralysis, or some evidence of involvement of the pulmonary, nervous, or gastrointestinal systems. Creighton had declared that there was something more than accident in the association between epidemics of influenza and epidemics of ague. Study of the last-named showed clearly that they were not, of course, outbreaks of malaria, but precisely those very gastrointestinal, pulmonary, and nervous manifestations which we are now beginning to realize actually constituted part and parcel of influenza prevalences.''

---

### SYMPTOMS.

A number of cases have been reported from the Hospital at

Galveston. A request to Dr. M. L. Graves for a symptomatic diagnosis brought the following answer.

Galveston, Texas, May 2, 1919.

Editor Texas Medical Journal:

Replying to your favor, I beg to advise that we have had several cases of lethargic encephaltitis, some seven or eight. Two of these have proved fatal after several weeks of profound sleep. No autopsies were obtained, but the cases appeared to be beyond doubt as to diagnosis.

Enclosed herewith you will find report of a case not yet fully recovered, but appearing to be convalescent at the present time. Man, sixty-six years of age; seen April 2, 1919. One week before the patient complained of numbness in the right hand and severe headache. Headache ceased. Patient complained of constipation; began to belch; complained of itching in hands and gaped constantly. Some difficulty in swallowing appeared. He became taciturn and depressed, not wanting to talk. Would discuss little business. He experienced some difficulty in moving right foot and there was diminished activity in the right hand, which difficulty continued to increase until the right hand became partially paralyzed and was freely movable by the patient with the left hand. He would stick out his tongue at times. The stupor began to deepen on the afternoon of April 2nd. Then he appeared greatly depressed and began to cry. The right leg and arm became more paralyzed. The superficial reflexes on the right side were absent. The right patellar reflexes were diminished. There was slight ptosis of the eyelids. A Kernig sign appeared in the left leg. A slight Babinski appeared in the right foot. There was no strabismus. The tongue was white and would project straight out of the mouth. By night the patient paid no attention to things about him apparently and showed no consciousness of pain. A slight difficulty in swallowing appeared. Drowsiness and stupor remained. The patient would open his eyes and would not speak; would soon close them and sink off into a deep lethargy. This continued until April 6th, when the Kernig in the right leg was more marked. The Babinski in the right foot was still present. Right arm and right leg still paretic; with no response to ordinary sense stimuli over the body. The stupor deepened. This condition remained about the same for four weeks, during which time the patient never spoke, but during the last week opened his eyes at times and looked around. He began to move the right foot at frequent intervals and then the right arm, with increasing evidence of recognition and practically complete subsidence of the stupor. The patient now appears to be convalescing and probably in time will entirely recover.

Trusting that this information will be helpful, and with kindest regards, I am,

Very truly yours,

M. L. GRAVES.

---

SEEING DOUBLE.

Merle, Texas, April, 21, 1919.

Texas Medical Journal:

In response to yours of recent date in regard to the sleeping sickness case I beg to describe as follows: Lula McG., colored school girl, age 13. About 16th March complained of slight headache and inability to see; as she explained at times: "I see two things (double vision). On 23rd of March her father consulted me, himself being sick, and also mentioned his girl being bilious and applied for medicine to move her bowels. On 27th of March received a call to come to house that the girl was sleeping and could not be awakened. Found her in bed, not emaciated but lying in one position; eyes muddy yellow and reddish; pupils normal, responding to light; bowels did not move for two days; glands could easily be palpated; tongue red and raw; temperature 101; urine suppressed; could awaken her by loud shouting or pressure on supra-orbital nerve; respiration 34-36. Cheyne-Stokes partly. While there kept her awake sufficiently to give her a good meal, but she went to sleep immediately after. Diagnosis, Sleeping Disease (Lethargic Encephalitis). At first auto-intoxication suggested itself, because so many come here suffering from biliousness, but this was dispelled by the clearing up of the bowels. Syphilis, shock and other things were excluded. On 28th used syringe to evacuate the bowels and patient felt better; on 29th appeared to be improving; on 30th could turn around and asked for something to eat. On April 1st received a hurry up call, patient's condition being worse, stayed from 8. p. m. to 1 a.m., condition growing worse; April 2nd patient in profound comatose condition, temperature rising to 104, pulse 140, breathing 60 short and jerky, continuing until 1:30 p. m. on 2nd, when she died. Treatment consisted of forced feeding, calomel every two hours, strichnia to support the heart which appeared faulty; advised not to leave her lying in one position for fear of bed sores developing. There was a consultation with another doctor, colored, who agreed in diagnosis and treatment.

As I am nine miles from a railroad and am used to all kinds of practice and conditions, a great many unusual cases come to my attention, but this was the first one of its kind, and I think I make no mistake in calling it a clear case of Lethargic Encephalitis or Sleeping Sickness, for she certainly did sleep.

DR. J. H. KOZAR.

---

### RECOVERY REPORTED.

Fort Smith, Arkansas, May 15, 1919.

Recent articles and reports of cases, especially those by Pothier, Jl. A. M. A. Mch. 8, 1919, and Bassoe, Jl. A. M. A. Apr. 5, 1919, and a special article in the same journal of March 15th seem to establish a train of symptoms characteristic of a new disease described under the head of "Sleeping Sickness," "Encephalitis Lethargica," or "Epidemic Encephalitis." A majority of the cases have followed Influenza and a majority of the reported cases have recovered.

Miss B., aged 23, a nurse in training, came to me Feb. 23, 1919.

Previous health good. She had a mild attack of Influenza late in January, 1919, but was off duty not longer than a week. About ten days before coming to me she had double vision at night when trying to read, which for the past two days had been constant.

She felt slightly drowsy and dull mentally.

Examination shows Homonymous vertical diplopia, image of right eye lowest, increased on looking down. Interpreted as paresis of the right inferior rectus. Eyes otherwise normal with vision 20/16 in each eye.

She was given ten gr. doses of iodide of soda and sent to bed.

Within two or three days she developed right facial paralysis and was seen by Dr. St. Cloud Cooper, who gave her some doses of Salicylate of soda. She remained slightly drowsy with slight fever, which was not above 100° F. In a few days drowsiness diminished and she became bright and mentally active. Two weeks from the time I saw her she was reported by Miss Tye, her superintendent, as entirely recovered of the diplopia and the facial paralysis, and she had returned to duty. No laboratory investigation was made. There was no reason to suspect Lues.

If one will read the reports referred to at the beginning of this communication he will agree that this case is quite as properly included as they under the same category.

H. MOULTON, M. D., F. A. C. S.

---

### ATAXIA SHOWN

Jeanerette, La., May 1, 1919.

Texas Medical Journal:

In answer to your inquiry as to a case of Sleeping Sickness in my practice, I take pleasure in giving you the following data:

Caroline Alexander, colored female, aged 48, married and mother of seven children. Consulted me April 10th, 1919. She had just returned from Port Arthur, Texas, where her daughter had died of Influenza. Complained of feeling very weak eyes red, palpitation of the heart, pulse 120 per minute, very drowsy, in fact could sleep day and night. Saw everything double (diplopia), fever 99 4-5 and at no time during her sickness did I find her with more than 100 fever. After the tenth day she developed ataxic symptoms, her right arm and right leg jerking almost continually, also the muscles of her face. There was marked constipation, dark brown coating on tongue, no headache at any time; urine specific gravity between 1014 and 1022; no albumen or sugar; acid reaction; difficulty of speech; heaviness of eyelids; expressionless face.

Gave her calomel and oil and kept bowels open with saline laxatives as needed. Arsenic (Fowler's Solution) in increasing doses, (daily). Iron, quinine and strychnine—Formin. Quieted nervous symptoms with bromides and chloral hydrate. Kept patient in bed, giving her liberal and nutritious diet. Her mind kept clear up to twenty four hours before her death, which took place on April 28th. Patient menstruated some time in May before falling sick, but no return up to her death. Hoping that this may be some little help.

Yours truly,

DR. S. N. PERRET.

